# A 100-km BOFDA Assisted by First-Order Bi-Directional Raman Amplification

**DOI:** 10.3390/s19071527

**Published:** 2019-03-29

**Authors:** Thomas Kapa, Andy Schreier, Katerina Krebber

**Affiliations:** Bundesanstalt für Materialforschung und -prüfung, Unter den Eichen 87, 12205 Berlin, Germany; andy.schreier@bam.de (A.S.); katerina.krebber@bam.de (K.K.)

**Keywords:** distributed Brillouin sensing, distributed temperature and strain sensing, BOFDA, stimulated Brillouin scattering, fiber optics sensors

## Abstract

We present, to our knowledge for the first time, a 100-km Brillouin Optical Frequency-Domain Analysis (BOFDA) employing a 200-km fiber loop. Compared to our previous publication, enhanced sensor length, sensor accuracy and spatial resolution are presented. The performance improvements are achieved by applying distributed Raman amplification (DRA) and a digital high-pass filter. We report on temperature measurements over sensing distances of 75 km and 100 km both with a 12.5-m spatial resolution. Temperature changes of 5 °C have been measured along 75 km sensing fiber. A temperature change of 30 °C has been detected at 99.5 km.

## 1. Introduction

Distributed Brillouin fiber optical sensing is used to measure strain and temperature, because of its linear relation to the Brillouin frequency shift (BFS). Distributed Brillouin sensing has been studied for several decades [1,2] in a wide range of applications. A common purpose is condition monitoring for large-scale infrastructures like dikes [3], pipelines [4], river embankments [5] and high voltage cables [6]. All long-range fiber sensors based on stimulated Brillouin scattering face similar challenges: (i) pump depletion, (ii) self-phase modulation and (iii) trade-off between spatial resolution, measurement uncertainty and sensor length. Brillouin Optical Time-Domain Analysis (BOTDA) has been considerably approved for decades [1,2] and provides sensing ranges about 100 km with a spatial resolution in a few meter-range [7,8]. State of the art BOTDA setups use techniques as 1st and 2nd order distributed Raman amplification [7,9,10], pulse codes [11], differential pulses [12], pre-pumped pulses [13], advanced image processing [14] and neuronal networks [15,16,17]. Recently, we reported on the first long-range BOFDA [18]. Compared to BOTDA, less investigations on BOFDA have been published [3,19,20,21,22,23,24]. However, BOFDA is a promising candidate to monitor lengths of several tens of kilometers with high spatial resolution.

In this paper, we report, to our knowledge for the first time, on a 100-km BOFDA for temperature monitoring with increased sensor length and improved spatial resolution and accuracy by using a 200 km fiber loop. The sensing fiber has a length of 100 km. To achieve this sensing fiber length, we used a digital high-pass filter [25] and distributed Raman amplification (DRA). It was shown that significant sensing range enlargements could be achieved by using DRA in BOTDA setups [7,8,26].

## 2. Experimental Setup

**Figure 1:**
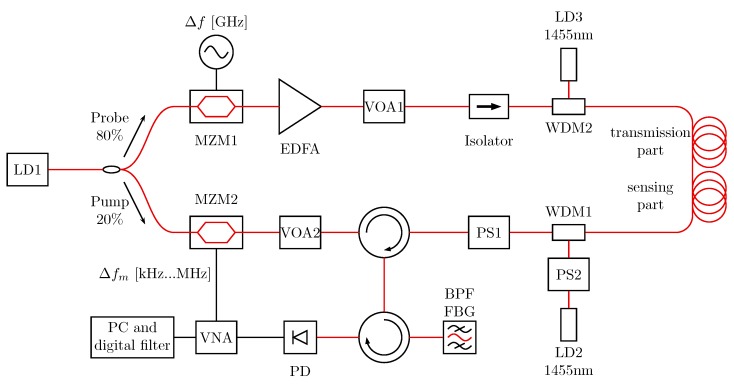
BOFDA sensor setup, LD: laser diode; MZM: Mach-Zehnder modulator; EDFA: Erbium-doped fiber amplifier; VOA: variable optical attenuator; PS: polarization scrambler; SMF: single mode fiber (for detailed configuration see Figure 2); BPF FBG: bandpass filter based on fiber Bragg grating; PD: photo diode; VNA: vector network analyzer, WDM: wavelength division multiplexer.

The measurement setup is shown in Figure 1. Compared to our previous setup [18], DRA at both ends of the fiber haul was implemented by 250 mW laser diodes at 1455 nm, respectively. Moreover, a polarization scrambler (PS) was implemented into the pump branch. Using DRA and due to the Raman-effect the wavelength of the light of the 1455 nm lasers was shifted to 1550 nm. The Raman-scattered light from the pump end amplifies the Brillouin scattering and the probe light, while the Raman-scattered light from the probe end amplifies the pump light. To minimize the dependency of different polarization of the Raman scattering light, pump and probe light, a second PS in one 1455 nm laser branch was used to guarantee a constructive interference of scattered light of 1455 nm with Brillouin scattering and probe light [7].

**Figure 2:**
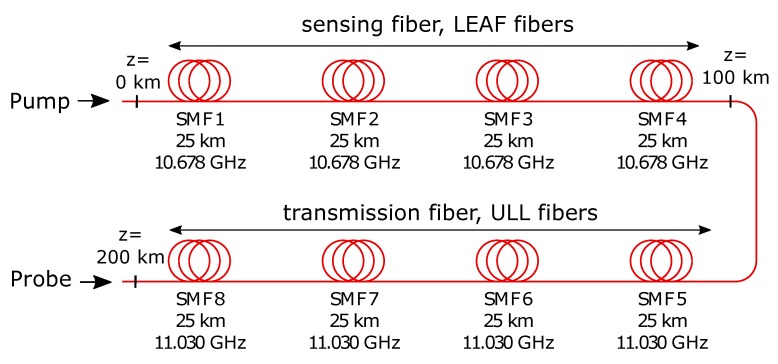
Configuration of the fiber loop (sensing and transmission fiber).

Figure 2 shows the configuration of the 200-km fiber loop. The sensing fiber of 100 km are Large Effective Area Fibers (LEAF) with a BFS of 10.678 GHz, the transmission fiber are Ultra Low Loss (ULL) fibers with a BFS of around 11.030 GHz. The second half of the fiber was used as transmission fiber. To avoid the Brillouin interaction in the transmission fiber (Brillouin frequency 11.030 GHz), the Brillouin gain spectrum (BGS) was measured around the Brillouin frequency of the sensing fiber (10.678 GHz). This measurement range ensured a stimulated Brillouin scattering (SBS) interaction only in the first half of the fiber haul and a higher pump power could be used [27].

For the 200-km fiber loop a minimum frequency of the VNA measurement was set to 500 Hz. The maximum freqency of the VNA measurement was set to 8.192 MHz for a spatial resolution of 12.5 m. The average count of the VNA measurement was set to 140. All transfer functions measured by the VNA were digital high pass filtered. Subsequently, the filtered transfer functions were inverse Fourier transformed to obtain spatially resolved backscatter traces. Together with additional time for data transfer and data processing, the measurement time was 18 h, caused mainly by narrow-band detection of the VNA. Due to long measurement time of BOFDA, the setup discussed in this paper can only be used for static measurements, where the measurement time is not relevant (e.g., long term geological changes, long term stability of structures, long term movements or hotspots of subsea power cables).

We carried out investigations over 75 km (in this case we removed SMF 4 and SMF 5 in Figure 2 from the fiber loop) as well as a 100-km sensing fiber. The power levels of all used laser sources for both sensing ranges are listed in Table 1, respectively. Pump and probe power are optimized to avoid pump depletion and to compensate loss of the additional fibers for 200 km fiber loop.

## 3. Experimental Results and Discussion

**Figure 3:**
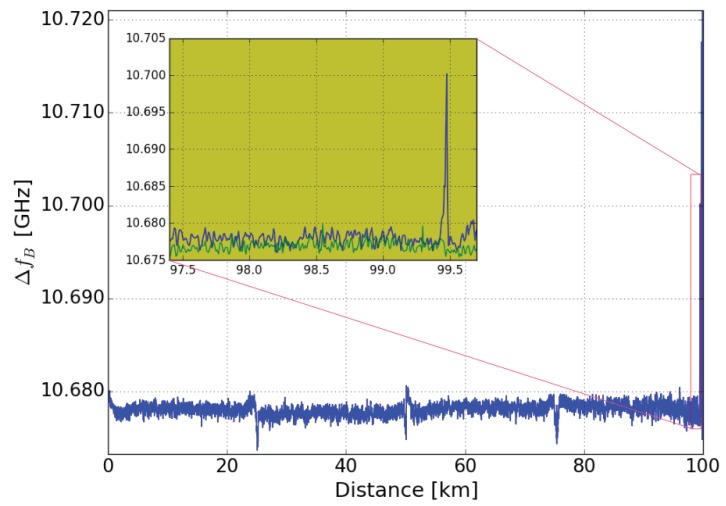
BFS over distance of 100-km sensing fiber, with 30 °C hotspot (blue), reference (green).

Figure 3 shows the BFS $∆f_{B}$ along the 100-km sensing part of the fiber. The four 25 km LEAF fibers can be well distinguished by the frequency dips of the connectors in between. At 99.5 km, 40 m were heated in a temperature chamber at 52 °C. The inset of Figure 3 provides a detailed view on the two BFS-measurements with (blue) and without (green) the local 30 °C-hotspot, respectively.

Room temperature was 22 °C, the spatial resolution was set to 12.5 m, $f_{B}$ was sweeped from 10.652 GHz to 10.724 GHz in steps of 6 MHz. The resolution bandwith of the VNA was set to 500 Hz. The 30 °C hotspot at 99.478 km was clearly detected.

**Figure 4:**
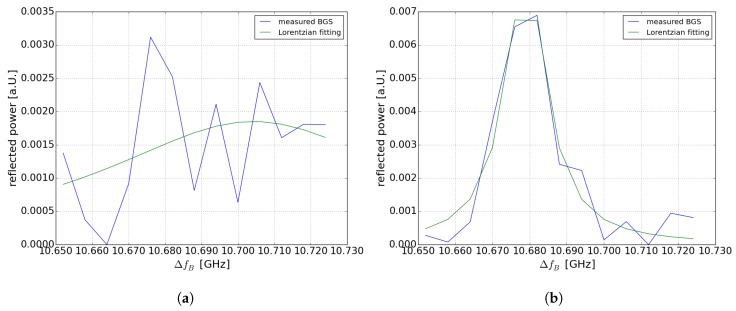
Reflected power of the 200-km fiber loop by using a digital high-pass filter (**a**) BGS at 99.478 km within the heated section (**b**) BGS at 99.521 km at room temperature.

**Figure 5:**
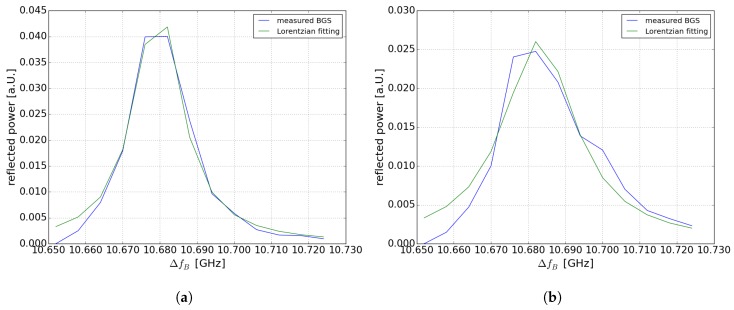
Reflected power of the 150-km fiber loop by using a digital high-pass filter (**a**) BGS at 74.200 km at room temperature (**b**) BGS at 74.156 km within the heated section.

Figure 4a,b show the BGS at room temperature at 99.521 km and at heated section at 99.478 km (30 °C). Figure 4a shows that at 10.678 GHz a “ghost” peak occurs. The “ghost” peak effect in BOFDA was discussed and explained in detail in [28]. Without “ghost” peak effect we should observe a shift of the Brillouin spectrum that should correspond to the 30 °C hotspot (30 MHz). As discussed in [28], the reason for the “ghost” peak effect is the interaction of the stationary component of the pump signal and the modulated acoustic wave. To remove this “ghost” peak a digital high-pass filter was used as presented in [25]. Nevertheless, in case of the 100-km sensing fiber this digital high-pass filter further decreases the already low SNR. This is why the value of the temperature at the location of the hotspot could not be exactly measured. However, it is possible to detect the hotspot at the corresponding location by using a Lorentzian fit of the Brillouin spectrum. In case of the 75 km fiber loop with higher SNR the temperature value can be measured, in comparison to the 100-km sensing fiber, where only a temperature change could be detected. In Figure 5b the Brillouin spectrum is shown after using the digital high-pass filter at the position of the hotspot. A distinction of the double peak is possible and the temperature value can be estimated. Even without a double peak fitting, the Lorentzian-fitted curve shifts in comparison with Figure 5a outside the heated section.

**Figure 6:**
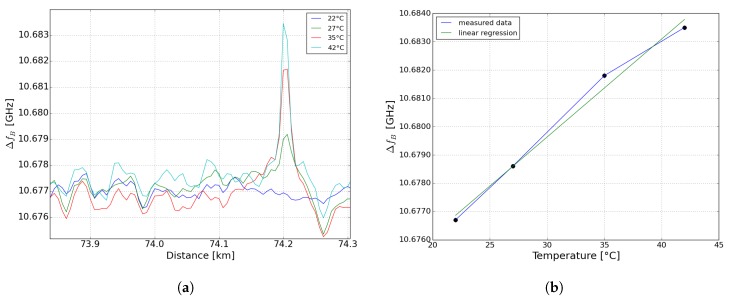
(**a**) Frequency shift at 74.2 km of a 40-m hotspot of 5, 13 and 20 °C (**b**) $∆f_{B}$ vs. temperature, 0.34 MHz/°C extracted by linear regression from Figure 6b.

The result of this single-peak fitting is shown in Figure 6a. The BFS at three different temperature values of the hotspot (5 °C, 13 °C and 20 °C) at 72.4 km of the sensing fiber are shown. The results were achieved by using the digital high-pass filter with a cutoff frequency of 250 kHz. By using linear regression, we have calculated a temperature coefficient of 0.34 MHz/°C (shown in Figure 6b). The measurement error compared to the linear regression is 0.4 MHz in maximum. There is a discrepancy between the calculated temperature coefficient of 0.34 MHz/°C and the standard temperature coefficient of approx. 1.2 MHz/°C of a LEAF fiber [29]. This is due to the “ghost” peak effect. However, still 5 °C could be measured (shown in Figure 6a).

**Figure 7:**
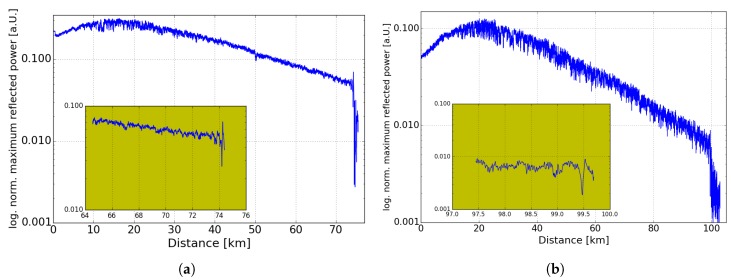
Logarithmized normalized maximum reflected power of the Brillouin gain spectrum (BGS) over distance of (**a**) 75 km sensing fiber with 20 °C hotspot (**b**) 100-km sensing fiber with 30 °C hotspot.

Figure 7a,b show the logarithmized normalized maximum reflected power of the BGS in case of a 75-km and 100-km sensing fiber scenario, respectively. Compared to reflected power profiles in BOFDA without DRA [18] a non-linear slope in logarithmic scale was observed, which is consistent to [7,9]. In Figure 7a,b there is a dip in the peak amplitude at 74.2 km and 99.478 km, caused by the broader BGS (shown in Figure 5b) at the heated sections. This dip can be used to reduce the measurement time by measuring only at one or some frequencies of the BGS [30,31]. At the end of the sensing fiber the level of the signal drops to the noise level. As mentioned above, in case of the 100-km sensing fiber there is a worse SNR compared to the 75-km measurement. However, the use of a digital high-pass filter leads to a detection of the hotspot in case of the 100-km. The high SNR in case of the 75 km enables a measurement of the temperature of the hotspot.

**Figure 8:**
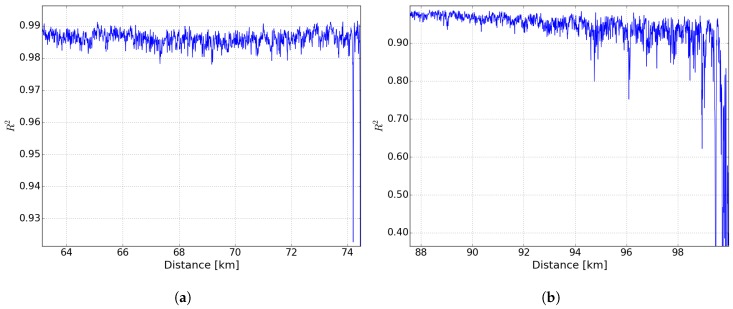
Coefficient of determination $R^{2}$, calculated by comparison of fitting with Lorentzian function and measurement data over distance for (**a**) 75 km and (**b**) 100-km sensing fiber.

Figure 8a,b shows the coefficient of determination $R^{2}$ of the Lorentzian fit in case of a 75 km and 100 km long sensing fiber. In case of a 75 km sensing fiber the $R^{2}$-values keep above 0.98 up to fiber end except for the heated section. In case of the 100-km a strong decrease of the $R^{2}$-values after 90 km is observed.

**Figure 9:**
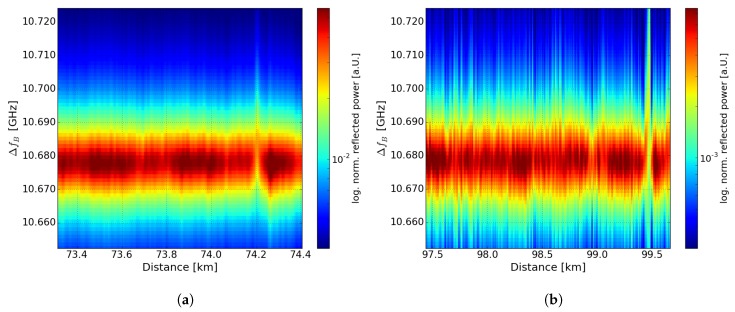
Color plot of logarithmized normalized reflected power of the Brillouin spectrum of a 40 m fiber section at (**a**) 74.2 km at a hotspot of 20 °C and (**b**) 99.48 km at a hotspot of 30 °C.

In Figure 9a,b a section of the Brillouin spectrum of the fiber loop is shown close to the position of the hotspot in case of a 75-km and 100-km long sensing fiber. The normalized reflected power is depicted in log-scale in order to improve the contrast of colors. At 74.2 km and 99.4 km the 40-m heated section of 20 and 30 °C could be clearly detected, respectively.

## 4. Conclusions

We demonstrate, to our knowledge the first time, a 100-km BOFDA for temperature monitoring by using 200 km fiber loop. A temperature change of 30 °C has been detected at 99.5 km with a spatial resolution of 12.5 m. In case of a 75 km sensing fiber a temperature change of 5 °C could be measured with a spatial resolution of 12.5 m. Compared to advanced time domain setups, potential improvement of the BOFDA setup is seen in image processing. Furthermore, the use of higher order Raman amplification and a laser with narrower linewidth could further reduce the noise, respectively. To enhance accuracy and reduce measurement time, neuronal networks could be used in future.

## Figures and Tables

**Table 1:** Pump, probe power and power of DRA lasers for 75 and 100 km sensing range.

Sensing Fiber Length (km)	Pump Power (μW)	Probe Power (μW)	Power of DRA (mW)
75	430	10	250
100	315	50	250

## Abbreviations

The following abbreviations are used in this manuscript:

BOFDA	Brillouin Optical Frequency-Domain Analysis
DRA	distributed Raman amplification
BFS	Brillouin frequency shifts
BOTDA	Brillouin Optical Time-Domain Analysis
SNR	signal-to-noise-ratio
WDM	wavelength division multiplexing
LD	laser diode
MZM	Mach-Zehnder modulator
EDFA	erbium-doped fiber amplifier
VOA	variable optical attenuator
PS	polarization scrambler
SMF	standard single mode fiber
FBG	fiber Bragg grating
BPF	band pass filter
PD	photo diode
VNA	vector network analyzer
LEAF	large effective area fiber
ULL	ultra low loss
BGS	Brillouin gain spectrum
SBS	stimulated Brillouin scattering
FoM	figure of merit
